# The oral microbiome is associated with stress, adversity, and mental health in young adults

**DOI:** 10.1038/s41598-026-60803-4

**Published:** 2026-07-13

**Authors:** Naomi N. Gancz, Paul W. Savoca, Bridget L. Callaghan

**Affiliations:** University of California, Los Angeles, USA

**Keywords:** Microbiome, Internalizing, Early adversity, Oral hygiene, Stress, Heart-rate variability, Diseases, Health care, Microbiology, Neuroscience, Psychology, Psychology, Risk factors

## Abstract

Acute stress responsivity and early-life adversity are both associated with oral microbiome differences that lead to increased mental and physical health risk. However, the *joint* association of stress responsivity and early adversity with the oral microbiome is not yet understood. Additionally, while the link between the oral microbiome and cortisol has been investigated, another major component of stress responsivity – the parasympathetic response – has been relatively neglected. Therefore, we examined parasympathetic functioning during an acute stressor, retrospective early adversity, oral microbiome (using the bacterial 16S gene), and anxiety and depression symptoms in 76 undergraduates. Early adversity was associated with lower microbiome diversity. Independently of adversity, parasympathetic withdrawal during the stressor was positively associated with *Alysiella*, a genus previously linked to latent viral infections. Early adversity and parasympathetic function did not significantly interact. Additionally, depressive symptoms were negatively associated with *Butyrivibrio*, which produces neuroprotective metabolites. Our findings suggest that childhood adversity and parasympathetic stress response are independently associated with oral microbiome differences, and that the oral microbiome is associated with mental health. By characterizing the associations of the oral microbiome with stress, adversity, and mental health, this study lays important groundwork for future research on psychophysiological causes and outcomes of oral microbiome differences.

**Supplementary Information:**

The online version contains supplementary material available at https://doi.org/10.1038/s41598-026-60803-4.

## Introduction

Exposure to chronic or severe stressors, especially early in life, is a risk factor for poor oral, physical, and mental health^1–4^. One factor that may play a role in the associations between stress exposure and health is the oral microbiome. In fact, the oral microbiota – the community of microorganisms that inhabit the oral cavity – can exert far-reaching effects not only on oral^5,6^ and systemic^7–9^ health outcomes, but also on the brain. Specifically, animal models have shown that oral microbiome dysregulation leads to increased biomarkers of neuroinflammation, as well as anxiety- or depression-like behaviors^9–11^. Further, in one study, it was found that transplantation of oral microbiota from chronic stress-exposed donor mice induces depressive-like behaviors in recipient mice^11^. Accordingly, in observational studies of humans, variations in oral microbiome composition have been linked to chronic stress^12–14^. The oral microbiome has also been linked to internalizing (anxiety or depression) symptoms in humans – this suggests that the effects of the oral microbiome on psychiatric health may translate from animal into human models^15–17^.

Although the effect of stress exposure on the oral microbiome has been established, the pathways by which the stress response affects the oral microbiome are not well-understood. Specifically, while in vitro^18–21^ experimental evidence and in vivo observational evidence^16,17,22^ support the effect of the *hormonal* arm of the stress response (i.e. cortisol) on the oral microbiome, the effect of the *autonomic* arm of the stress response (i.e. parasympathetic and sympathetic nervous system change, typically measured through peripheral electrophysiology) on the oral microbiome is largely unexplored. However, evidence suggests that the interindividual differences in autonomic function (i.e., tone or reactivity to stimuli) may be important contributors to oral microbiome composition: The parasympathetic branch of the autonomic nervous system modulates saliva flow rates^23^, which can affect the oral microbiome community^24^, and the sympathetic branch releases salivary catecholamines^4^ that may alter bacterial growth rates^21,25^. Moreover, both branches induce the release of starch-degrading, antibacterial salivary proteins^23,26,27^. Given the evidence that autonomic function may be associated with oral microbiome composition, the lack of research on the link between these systems comprises a critical research gap. Characterizing the associations between oral microbiome composition and autonomic function is therefore necessary for a more complete understanding of the relationship between stress exposure and the oral microbiome.

Additionally, the type and timing of stress exposure likely matters for oral microbiome composition. Specifically, early interpersonal adversity (IA; significant separation from or maltreatment by a caregiver or other close individual during childhood), a highly impactful type of stressor^28^, may have a unique relationship with the oral microbiome – for example, the oral microbiomes of adults with a history of higher IA have decreased potential for tryptophan degradation and glutamate synthesis^29^. Further, young adults with a history of IA show decreased abundance of oral microbiota that are linked to lower immune system aging^30^. In youth, IA exposure is linked to lower abundance of largely commensal oral microbiota, and slightly increased abundance of internalizing symptom-linked potential pathogens^17^.

Interestingly, IA in early life is associated with differences in how the oral microbiome responds to stress exposure later-in-life^17^. For example, hair cortisol (which indexes average cortisol release over the past month) is negatively associated with oral microbiome richness in youth without IA exposure^17^. This association is blunted in IA-exposed youth, suggesting that IA moderates relationships between cortisol (part of the hormonal arm of the stress response) and the oral microbiome^17^. This prior research aligns with the stress sensitization model^31^, which posits that IA may potentiate vulnerability of certain biological systems to challenges, such as acute stress, later in life. However, more research is needed to examine whether associations between different stress components (e.g. parasympathetic reactivity to stressors) and the oral microbiome also aligns with the stress sensitization model. In other words, insufficient research has examined how IA and non-hormonal components of the stress response relate to the oral microbiome, either independently or in interaction with each other, nor the relevance of these processes for mental health.

In sum, while the literature supports a link between psychosocial stress and oral microbiome dysregulation, the mechanisms underlying this effect remain poorly understood, including the role of the autonomic nervous system, the additive or moderating role of IA, and the relevance of stress-related oral microbiome differences for mental health. The present study seeks to address these issues by examining electrophysiological measures of parasympathetic activity at baseline and during an acute stress task, history of IA exposure, oral microbiome composition, and anxiety and depression symptoms in *N* = 86 undergraduates (with a final sample size of *N* = 76 with quality data). Although both the sympathetic and parasympathetic arms of the autonomic nervous system are likely relevant to the oral microbiome, the present study only examines the parasympathetic arm. We chose to focus on the parasympathetic arm, rather than both arms, to reduce participant burden caused by placement of additional electrodes, and because we had previously found that, in our lab, electrophysiological measurements of parasympathetic activity led to cleaner signal and therefore minimized data loss compared to measurements of sympathetic activity.

## Methods

### Participants

Eighty-six undergraduate students at the University of California, Los Angeles participated in the study for course credit (see Table 1). Inclusion criteria were being at least 18 years old and fluent in English; exclusion criteria were having uncorrected vision or hearing problems, or having felt ill, taken antibiotics, or taken antifungals within the month prior to participation. The sex and ethnicity distribution of our sample was typical of studies of undergraduates participating for credit at the University of California, Los Angeles (see Table 1). One participant did not complete the oral hygiene questionnaire and was thus excluded from all analyses except the correlation matrix. This study was approved by the University of California, Los Angeles institutional review board (IRB#20–000295). Procedures were performed in accordance with all relevant guidelines and regulations. Informed consent was obtained from all participants.

**Table 1:** Sample demographics.

Variable	Mean (SD)	Cronbach’s Alpha
*Age*	21.01 (3.62)	
*BDI*	9.48 (8.14)	0.91
*STAI-T*	41.92 (10.20)	0.93
*CTQ*	87.83 (12.2)	0.92
**Variable**	**Count (Proportion)**	
*Sex*		
Female	60 (69.77%)	
Male	26 (30.23%)	
*Ethnicity*		
East Asian	23 (26.74%)	
White	19 (22.09%)	
Multiple/Other	17 (19.77%)	
South Asian	11 (12.79%)	
Latinx/Hispanic	9 (10.74%)	
Black/African American	4 (4.65%)	
Middle Eastern	3 (3.49%)	

This table shows the means and standard deviations of age, Beck Depression Inventory (BDI)^35^, State Trait Anxiety Inventory – Trait (STAI-T)^36^, and Childhood Trauma Questionnaire (CTQ)^34^. Cronbach’s Alpha (calculated after item exclusion in the case of CTQ) is shown where calculated. For sex and ethnicity, counts and proportions are shown.

### Procedure

Upon arriving at the laboratory, participants were asked to refrain from consuming any food or drink for 30 min until saliva sample collection. Participants then completed questionnaires, after which a trained researcher measured their height and waist circumference. Then, with guidance from a researcher, participants collected saliva samples for oral microbiome sequencing using *OMNIgene•ORAL* kits (DNA Genotek). Subsequently, for the electrophysiology portion of the study, participants were seated in a comfortable chair as researchers prepared electrode placement sites with an abrasive gel, then placed disposable electrocardiography (ECG) electrodes in a modified Lead II configuration using a conductive gel to ensure good contact with the skin. A respiration belt was also placed just below the participant’s sternum. ECG and respiration were then recorded using a BIOPAC BioNomadix BN-RSPEC module, with a sampling rate of 1000 Hz.

For the 200-second baseline block, participants viewed Inscapes, a validated, restful video of abstract geometric shapes with calming music^32^. Then, participants completed the Montreal Imaging Stress Task (MIST)^33^, which begins with a 2-minute practice of untimed, difficult arithmetic problems, followed by 2, 2-minute runs of timed problems (comprising the stress block), during which participants were told their performance was considerably below average. Between the stress blocks, a researcher reminded participants that their performance was below average. This task varied slightly from the original publication in the duration of blocks, the visual appearance of the screen and user interface, and the mathematical problems used. Finally, participants watched Inscapes again for the 200-second recovery block (Supplement 1).

### Questionnaires

IA was assessed using the Childhood Trauma Questionnaire (CTQ)^34^ with the item, “I didn’t have enough to eat” removed as it is not necessarily reflective of IA^28^ – reliability remained high even after removal of this item (Table 1). Additionally, depressive symptoms were assessed using the Beck Depression Inventory (BDI)^35^, anxiety symptoms using the State Trait Anxiety Inventory – Trait (STAI-T)^36^, diet quality using the Rapid Eating Assessment for Participants – Short (REAP-S)^37^, chronic stress using the Perceived Stress Scale (PSS)^38^, and oral hygiene using a scale adapted from Buunk-Weerkhoven et al.^39^, with the brushing method item not measured due to time constraints and the fluoride item not included due to high missingness. We decided a priori that when 30% or fewer item responses were missing within a questionnaire, they would be imputed to that participant’s mean response value. We found that no questionnaire had more than 1 missing item needing to be imputed. Where appropriate, questionnaire measures that were highly skewed were natural log- or square root-transformed to ensure that assumptions of normal distributions of residuals were met. Finally, all questionnaire measures were standardized and centered at the mean.

One participant did not complete the oral hygiene questionnaire and was thus excluded from all analyses including that scale.

### Heart-Rate Variability Pre-Processing

We used NeuroKit2^40,41^ to identify R-peaks, which were visually inspected by trained researchers. Where artifacts prevented the identification of a clear R-peak, one was interpolated halfway between adjacent peaks. All epochs were of sufficient quality to be retained. Then, High Frequency-Heart-Rate Variability (HF-HRV; HRV in the 0.12–0.4 Hz frequency band) was calculated within each 30-second epoch using NeuroKit2^40^. HF-HRV is a proxy for respiratory sinus arrhythmia, which represents parasympathetic input to the heart, and it is a widely accepted and validated measure of parasympathetic activity^42^. The selection of a 0.12–0.4 Hz frequency band was derived from published guidelines for HF-HRV studies^42^. Blocks with respiration rate outside the 0.12–0.4 Hz frequency band were discarded. Baseline HF-HRV (HF-HRV) was defined as the average HF-HRV during the first minute of the baseline, HF-HRV reactivity was defined as the change in average HF-HRV from the first minute of the baseline to the first minute of the stress task, and HF-HRV recovery was defined as the change in average HF-HRV from the first minute of the stress task to the last minute of the recovery block. The decision to use the first minute of each block was made to avoid effects of habituation to or distraction from stimuli and is consistent with previous research^43,44^. HF-HRV was natural log-transformed and standardized.

Seven participants were excluded from HF-HRV analyses due to technical issues resulting in data being unusable. Additionally, due to respiration being outside the 0.12–0.4 Hz frequency band, 2 participants were excluded from the baseline block, 8 from the stress block, and 2 from the recovery block, resulting in a total *N* = 77 participants with useable data for baseline HF-HRV, *N* = 71 for HF-HRV reactivity, and *N* = 70 for HF-HRV recovery.

Low frequency HRV was not calculated as evidence suggests that it may not be a good measure of sympathetic activity^42^, and thus we had no a priori hypotheses about its relationship to the oral microbiome.

### 16S rRNA microbiome sequencing and pre-processing

After collection, saliva samples were aliquoted following manufacturer instructions and stored at −80 °C until being transferred to the UCLA Microbiome Core facility on dry ice. DNA was extracted by bead beating using MAG MAX 96 well-plate kits paired with a Thermo KingFisher (Waltham, MA) according to the manufacturer’s instructions. The V4 region of the 16S rRNA gene was amplified using barcoded primers containing Illumina flow-cell compatible adapter sequences. The 16S gene has been widely used for microbiome sequencing because it is conserved among all bacteria, while varying sufficiently between taxa to be used for taxonomic identification at the genus level; it is not present in host DNA^45^ The resulting amplicons were pooled in equal amounts to create the sequencing library, which was sequenced on an Illumina MiSeq using the 2 × 250 bp v2 kit.

The sequences were preprocessed using Qiime2 software, version 2024.10^46^. Specifically, denoising and estimation of amplicon sequence variants (ASVs) were conducted using DADA2^47^, a phylogenetic tree constructed using FastTree^48^, and taxonomic assignment conducted using a bespoke Naïve Bayes classifier trained with a k-mer length of 12^49,50^. Data were rarefied to a sequencing depth of 21,109, which allowed all samples to be retained. We then calculated Faith’s alpha diversity^51^, which captures richness of the microbiome while weighting for phylogenetic relatedness, and Shannon’s alpha diversity, which incorporates both richness and evenness^52^ Finally, estimated functional pathways of the microbiome based on taxonomic data were calculated using the PICRUSt2 pipeline^53^.

Next, the genus abundance table and the PICRUSt2 estimated pathway abundance table were each filtered to retain only features present in at least 20% of samples, then center log-ratio transformed using the MaAsLin2 pipeline^54^. We then further filtered the genus and PICRUSt2 pathway abundance tables to select for an “oral-brain module” (Supplement 3, Table S1): a set of genera and pathways that could potentially affect the brain based on human and animal literature, similar to the previously proposed “gut-brain module”^55^. The oral-brain module was used to improve power by limiting the number of statistical comparisons.

In sum, we examine 3 oral microbiome measures derived from 16S sequences: alpha diversity, genus-level composition, and estimated functional pathways. These represent 3 different oral microbiome characteristics which could interact with host behavior and physiology.

### Covariates

All analyses controlled for sugar consumption (a sum of the REAP-S^37^ sweetened food and sweetened drinks items) and oral hygiene quality. Additionally, analyses examining HF-HRV reactivity or recovery controlled for baseline HF-HRV.

To preserve statistical power, our analyses did not control for chronic stress (PSS) or adiposity (waist to height ratio), as our supplemental analysis found that they were not significantly associated with oral microbiome composition (Supplement 2; Tables S2-S3).

We did, however, find that time of day of study participation was significantly associated with Shannon’s alpha diversity (Supplement 3; Table S4). Therefore, for significant results involving Shannon’s diversity, we conducted sensitivity analyses to test if the association was robust to controlling for time of day (Supplement 3; Table S4).

### Statistical analyses

Statistical analyses were conducted using R software 4.2.1.

#### Effectiveness of stress manipulation

Effect of the stress manipulation was tested using a hierarchical linear model with HF-HRV as the outcome and block (baseline, stressor, or recovery) as a level-1 predictor.

#### Correlation matrix

To examine the correlation structure among non-microbiome variables and covariates (including those not ultimately included in primary analyses), we constructed a matrix of Pearson’s correlations for each pairwise combination of variables.

#### IA, parasympathetic function, and the oral microbiome

We used ordinary least squares (OLS) regressions to test whether the interaction of IA * Baseline HF-HRV, as well as main effects of IA and baseline HF-HRV, were associated with alpha diversity metrics. We repeated this for HF-HRV reactivity and HF-HRV recovery. We used Benjamini-Hochberg (BH) multiple comparison correction with a false discovery rate (FDR) threshold of 0.250, which is recommended for exploratory analyses^56^, on the explanatory variables of interest. Because the 3 HRV metrics were expected to be correlated, each was included in a separate “family” of models – a separate group of models for the purpose of multiple comparison correction. We determined a priori that in cases where an interaction effect was not significant for any model within a family of models, those models would be re-run with the interaction effect removed in order to ensure that models are correctly specified. Otherwise, if the interaction effect was significant for any model within a family of models, it was retained for all models.

We repeated this process for transformed and filtered genera and PICRUSt2 features, such that there were 6 families of models in total: one for each of the 3 HF-HRV metrics, for both genera and PICRUSt2 features.

#### Oral microbiome and anxiety and depressive symptoms

Next, we ran a set of OLS regressions with microbiome features present in the oral-brain module as the explanatory variables and anxiety and depression symptoms as the outcome variables, controlling for covariates, IA, and Baseline HF-HRV (to avoid confounding effects of parasympathetic function). Each type of microbiome feature (alpha diversity, genera, and PICRUSt2 pathways) and type of symptom comprised a family of models for multiple comparison correction, yielding 6 model families, again using BH correction on the explanatory variables of interest with an FDR threshold of 0.250.

#### Oral microbiome and health behaviors

We used OLS models to separately test the associations of sugar consumption or oral hygiene with oral microbiome features, controlling for IA and Baseline HF-HRV. Each type of microbiome feature (alpha diversity, genera, and PICRUSt2 pathways) and type of health behavior comprised a family of models for multiple comparison correction, yielding 6 model families, again using BH correction on the explanatory variables of interest with an FDR threshold of 0.250.

### Power analysis

We conducted a *post-hoc* power analysis using GPower software. Although the actual critical p-value threshold is not known a priori until it is calculated during the FDR correction, we chose a threshold of *p* <.01 as an approximation. With our sample size of *N* = 76 (accounting for excluded data), a model with 5 predictors (e.g., IA * HF-HRV with main effects and covariates) has 59.75% power to detect an f^2^ effect size of 0.11. This effect size was derived from our findings in a similar study^17^. The power to detect a medium effect size of 0.15 is 76.43%.

## Results

### Effectiveness of stress manipulation

**Fig. 1:**
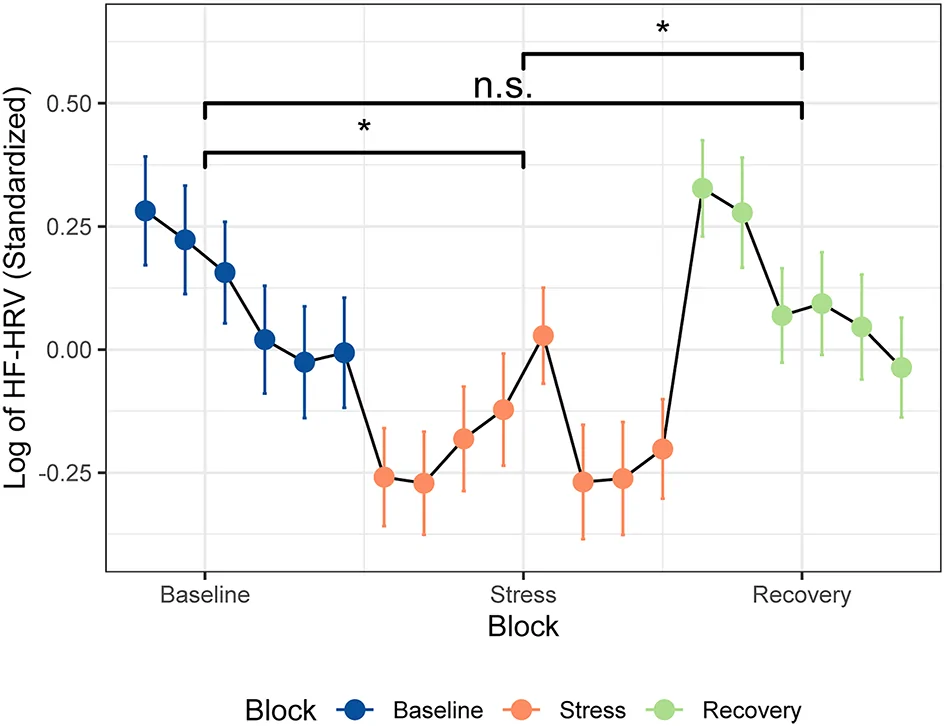
The standardized natural log of HF-HRV is shown on the Y axis and time is shown on the X. Each circle represents average HF-HRV within a 30-second epoch and error bars show the standard errors. Blocks are shown by colors. Brackets at the top show “*” for significant or “n.s.” for nonsignificant differences between blocks.

Confirming the effectiveness of the stress manipulation, HF-HRV during the stress task was significantly lower than both baseline (b = −0.33, t (1415.07) = −9.66, *p* <.000) and recovery (b = −0.37, t (1415.46) = 11.06, *p* <.000), but the baseline and recovery blocks did not significantly differ from each other (b = 0.05, t (1414.74) = 1.36, *p* =.173; Fig. 1).

### Correlation matrix

**Fig. 2:**
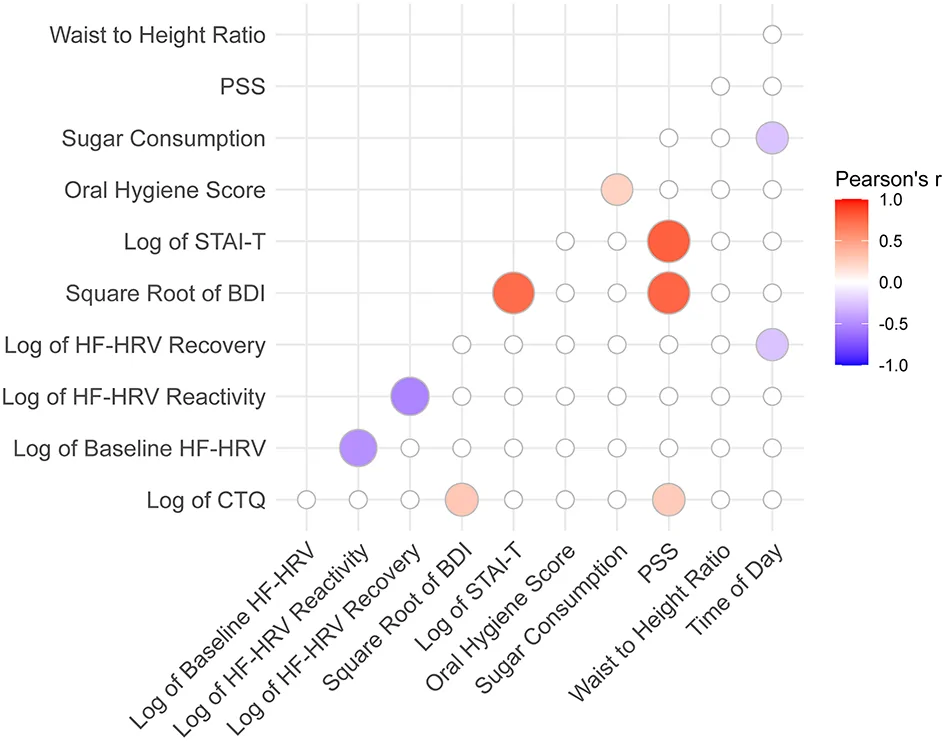
Pairwise correlations are shown, wherein significant positive correlations are shown as red circles and negative correlations as blue circles; larger circles indicate stronger correlations. Nonsignificant correlations are shown as small white circles.

Several questionnaire measures were significantly intercorrelated, as were HF-HRV measures (Fig. 2, Table S5).

### IA, parasympathetic function, and the oral microbiome

We tested whether IA and parasympathetic function were linked with oral microbiome composition, either jointly or in interaction with each other. We found that IA and parasympathetic function did not significantly interact in any model (Tables S6-S8). Thus, the interaction term was removed from all analyses.

**Fig. 3:**
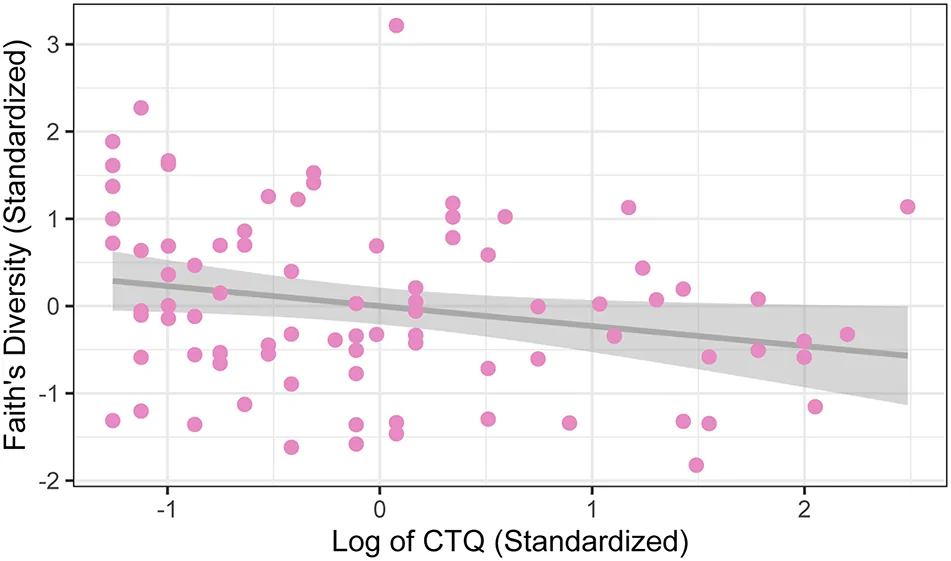
Standardized Faith’s Diversity is shown on the Y axis and the standardized natural log of CTQ on the X. A dark gray line shows the bivariate line of best fit, and lighter gray shading shows 95% confidence intervals.

While there was no significant interaction effect, IA and parasympathetic function did independently associate with oral microbiome features: IA was significantly negatively associated with Faith’s alpha diversity when controlling for HF-HRV baseline (b = −0.25, t (71) = −2.18, q = 0.129), reactivity (b = −0.25, t (65) = −2.08, q = 0.166), or recovery (b = −0.25, t (64) = −2.09, q = 0.162; Tables S9-S11; Fig. 3). IA was not significantly associated with other microbiome features.

**Fig. 4:**
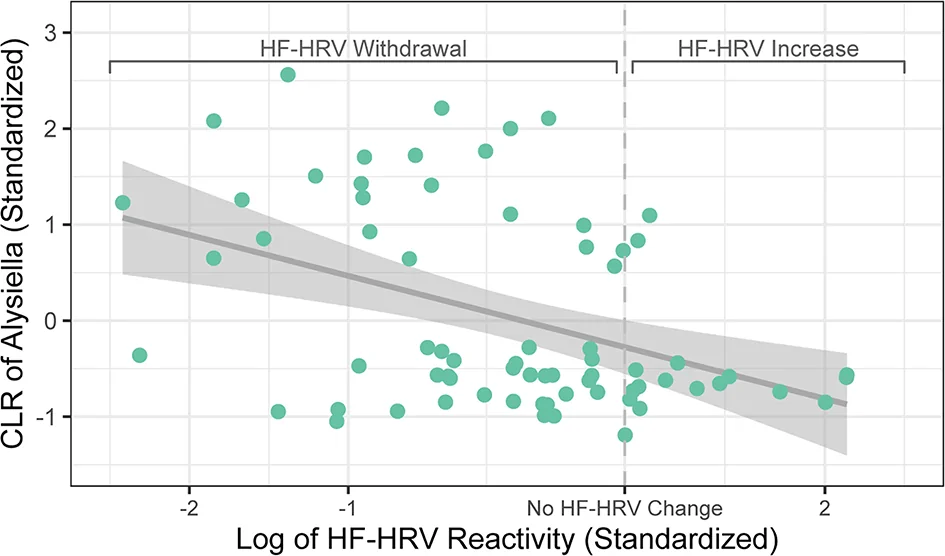
Center log-ratio transformed *Alysiella* relative abundance is shown on the Y axis and the standardized natural log of HF-HRV reactivity to the stress task on the X. A dark gray line shows the bivariate line of best fit, and lighter gray shading shows 95% confidence intervals. A dotted vertical line indicates no HF-HRV change, while values to its left indicate HF-HRV withdrawal during stress, and values to the right indicate HF-HRV increase.

At the genus level, HF-HRV reactivity was significantly negatively associated with *Alysiella*, a bacterial genus in the *Neisseriaceae* family (b = −0.42, t (65) = −3.12, q = 0.237; Table S10; Fig. 4), when controlling for IA. HF-HRV reactivity was not significantly associated with other microbiome features, and baseline and recovery HF-HRV were not significantly associated with any microbiome features.

Additionally, there were no significant associations between any explanatory variable of interest and PICRUSt2 features (Tables S9-S11).

### Oral microbiome and anxiety and depression symptoms

**Fig. 5:**
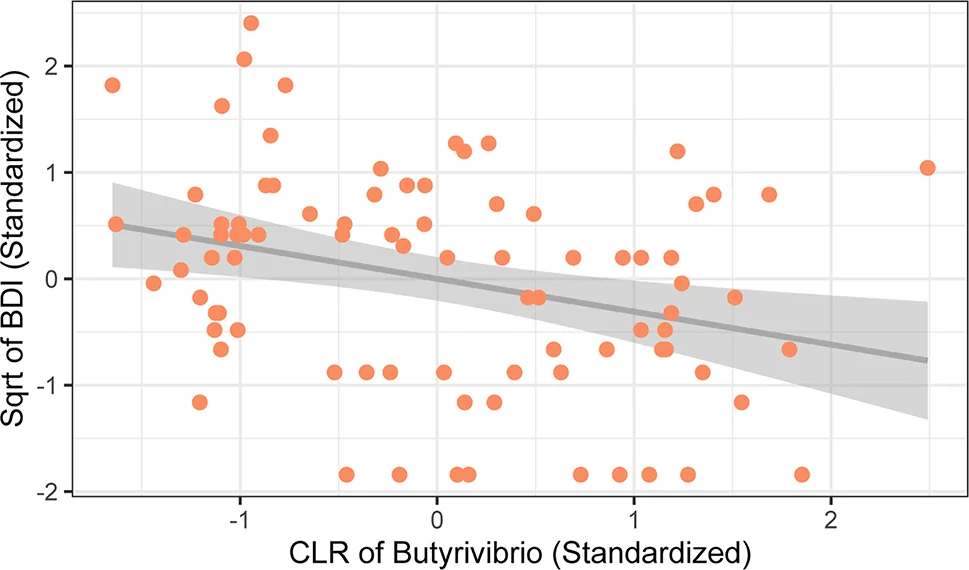
Standardized, square root-transformed BDI is shown on the Y axis and center log-ration transformed *Butyrivibrio* abundance on the X. A dark gray line shows the bivariate line of best fit, and lighter gray shading shows 95% confidence intervals.

Next, we tested whether any oral microbiome features were associated with anxiety or depressive symptoms. We saw that depressive symptoms were significantly negatively associated with *Butyrivibrio*, a bacterial genus in the *Lachnospiraceae* family (b = −0.33, t (70) = −3.28, q = 0.072; Fig. 5). Depressive symptoms were not significantly associated with any other microbiome features, and anxiety symptoms were not significantly associated with any microbiome features (Tables S12-S13).

### Oral microbiome and health behaviors

**Fig. 6:**
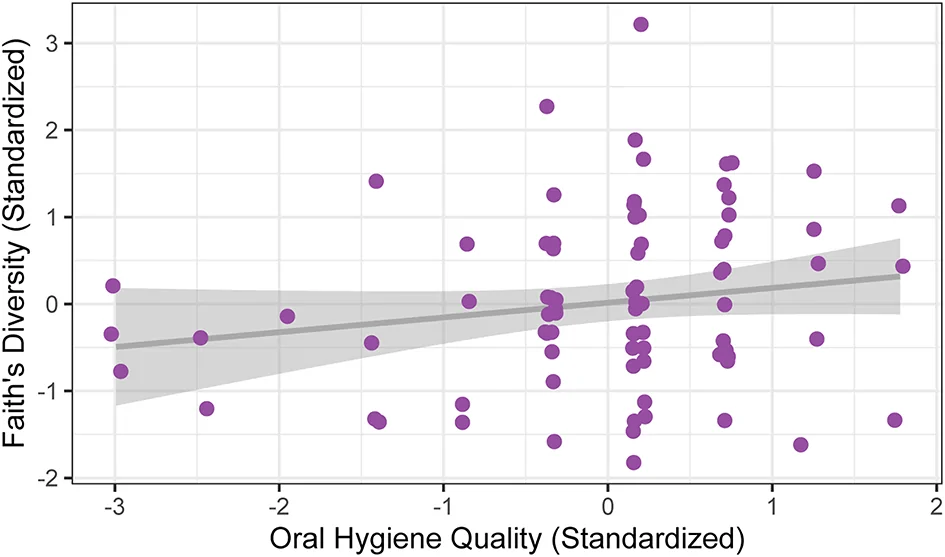
Standardized Faith’s Diversity is shown on the Y axis and standardized oral hygiene quality on the X. A dark gray line shows the bivariate line of best fit, and lighter gray shading shows 95% confidence intervals. Points are slightly jittered along the X axis for improved visibility.

Finally, to examine the relationship between health behaviors – sugar consumption and oral hygiene – and the oral microbiome, we examined these behaviors as predictors of interest, rather than nuisance variables. Oral hygiene quality was significantly positively associated with Faith’s alpha diversity (b = 0.22), t (70) = 2.01, q = 0.143; Fig. 6), but no other microbiome features (Table S14). Sugar consumption was not significantly associated with any microbiome features (Table S15).

## Discussion

Evidence suggests that the effects of chronic or severe stressors on the oral microbiome may be relevant for stress-induced mental health risk. However, the relationship between the autonomic arm of the stress response, as well as early-life IA, and the oral microbiome are not well-understood. In this study, we sought to examine how autonomic (parasympathetic) responsivity to acute stress relates to the oral microbiome jointly with IA, following the stress sensitization framework, as well as the extent to which oral microbiome features explain variance in depression and anxiety symptoms.

We found that self-reported IA and parasympathetic function did not significantly interact to explain variance in the oral microbiome, and that they were independently associated with oral microbiome features. Specifically, participants with higher self-reported IA had lower Faith’s alpha diversity, meaning that they had fewer phylogenetically distinct variants of bacteria. Lower Faith’s diversity has previously been associated with risk factors such as depressive symptoms and chronic stress^12,57–59^, although this finding has not been consistent across the literature^11,16,29,60^. On the other hand, parasympathetic function was not significantly related to any alpha diversity metrics; rather, those with higher HF-HRV withdrawal during acute stress had higher relative abundance of the bacterium *Alysiella*, a common oral microbe that has previously been positively associated with immune system aging biomarkers^30^. In sum, while both IA and parasympathetic stress task reactivity were associated with oral microbiome features that have elsewhere been linked to mental and physical health risk, these associations were independent of each other, suggesting that IA relates to the oral microbiome through different pathways than does the acute autonomic stress response in adulthood. Thus, our results do not provide evidence that links between IA, parasympathetic activity, and the oral microbiome are aligned with the stress sensitization framework. Further, the health implications of both Faith’s diversity and *Alysiella* require further research.

Indeed, while both Faith’s diversity and *Alysiella* were associated with IA and parasympathetic stress task reactivity, respectively, neither of these was associated with anxiety or depressive symptoms in the present study. However, depressive symptoms were negatively associated with relative abundance of *Butyrivibrio*. This could potentially be explained by the fact that *Butyrivibrio* ferments fibers into short-chain fatty acids^61^, a metabolite that is beneficial for brain health^62^. It should be noted, however, that unlike in the gut, where short-chain fatty acid production is largely beneficial, *oral* short-chain fatty acids can facilitate disease risk^62^. Therefore, this finding should guide future research into the role of short-chain fatty acid-fermenting bacteria in the relationship between the oral microbiome and brain.

Although we treated health behaviors – oral hygiene quality and sugar consumption – as nuisance variables in most of our models, we also examined their associations on their own. While sugar consumption was not significantly related with oral microbiome features, oral hygiene quality was positively associated with Faith’s alpha diversity. In the literature, the association between oral hygiene and alpha diversity is mixed, possibly due to heterogeneity in the age and oral health status of study populations^63–65^. Nonetheless, the fact that, in the present study, the effect size of the positive association between Faith’s diversity and oral hygiene was similar to the effect of the negative association between Faith’s diversity and IA should encourage future research to more closely examine whether oral hygiene quality could counteract harmful effects of IA on the oral microbiome.

Considered together, our findings show that both IA and parasympathetic stress task reactivity are associated with oral microbiome characteristics that may indicate increased health risks, but that these associations are independent of each other, suggesting they relate to the oral microbiome via distinct pathways. Further, we find that *Butyrivibrio*, a bacterium fermenting fiber into short-chain fatty acids, is negatively associated with depressive symptoms, and that Faith’s diversity is positively associated with oral hygiene, suggesting that fiber-fermenting bacteria and oral hygiene should both be further examined as potential intervention targets for improving oral microbiome and psychiatric health, especially in the context of recent or early-life stress.

Notably, our effect sizes are modest and limited to a small number of oral microbiome features. This speaks to the fact that, especially in young adults, the oral microbiome is fairly resilient against risk factors, as helpfully reviewed by Wade (2021)^66^.

Additionally, it should be noted that the oral microbiota comprise a complex ecosystem that is influenced by context, behavior, and physiology, rather than being a direct readout of distress or mental health.

### Limitations

Although this study provides important evidence for the associations between the oral microbiome and parasympathetic stress task reactivity, early IA, and depressive symptoms, several limitations should be addressed in future research: our convenience sample of undergraduate students is not representative of young adults in the United States, and thus our findings are not generalizable to that population. Additionally, although 16S sequencing is a low-cost and efficient method of microbiome sequencing that is suitable for nascent fields, such as the study of relationship between the oral microbiome and brain, higher-cost sequencing types, such as shotgun sequencing, are necessary to more precisely estimate bacterial functions and strain-level microbiome composition. Further, although we collected high-quality ECG data, we were unable to collect measures, such as pre-ejection period or salivary catecholamines, enzymes, or cortisol, that would capture other facets of autonomic or endocrine function that may be relevant for oral microbiome composition. This is of particular importance given that HF-HRV reflects parasympathetic input to the heart, which may differ from parasympathetic input to oral tissue. In spite of these limitations, the findings of this study lay the groundwork for future research on the mechanisms underlying the relationships between the oral microbiome, stress, adversity, and mental health.

## Conclusion

Parasympathetic stress task reactivity and early IA are independently associated with oral microbiome features that may indicate increased health risk. However, fiber-fermenting bacteria and high-quality oral hygiene may reduce oral microbiome-derived health risks. Future research should examine broader populations, employ advanced microbiome sequencing techniques, and measure different products of the stress response to better characterize the psychophysiological sources and consequences of oral microbiome dysregulation.

## Supplementary Information

Below is the link to the electronic supplementary material.

Supplementary Material 1

Supplementary Material 2

## Data Availability

The datasets generated and/or analysed during the current study are available in the SRA repository, accession number PRJNA1431472 (https://www.ncbi.nlm.nih.gov/sra/PRJNA1431472).
