# Supplementary material for: The oral microbiome is associated with stress, adversity, and mental health in young adults
Source: Sci Rep. 2026 Jul 13;16:32157. doi: 10.1038/s41598-026-60803-4 (PMC13661280; doi:10.1038/s41598-026-60803-4)
Supplement: Supplementary file 1 [file 41598_2026_60803_MOESM2_ESM.docx]

**Supplement 1: Study Procedure**

**Supplement 2: Oral-Brain Module**

The oral-brain module includes genera that are represented in the major subgingival microbial complexes^1^ (groups of interdependent or co-occurring bacteria linked to biofilm formation), known to produce hydrogen sulfide^2^, known to reduce nitrate^3,4^, potentially caries-related^5^, previously associated with in-vivo or in-vitro levels of cortisol^6,7^, or previously associated with self-reported stress or internalizing symptoms in humans^6,8–14^. The oral-brain module also includes pathways, referenced with the MetaCyc database^15^, that might be relevant to the brain, including those involved in synthesis or degradation of aromatic amino acids (e.g. tryptophan)^16^, short-chain fatty acids^16^, hydrogen sulfide, nitrate^3,4^, lipopolysaccharide^17^, peptidoglycan^16^, gamma-aminobutyric acid (GABA)^18^, or glutamate^16^.

| **Table S1: Oral-Brain Module** | | |
| --- | --- | --- |
| **Taxonomic Features** | | |
| *Function/Description* | *Genera* | *Citation(s)* |
| Members of the major subgingival microbial complexes | *Actinobacillus; Actinomyces; Campylobacter; Capnocytophaga; Eikenella; Eubacterium; Fusobacterium; Peptostreptococcus; Porphyromonas; Prevotella; Selenomonas; Tanerella; Treponema; Veilonella* | Mohanty et al., 2019^19^ |
| Potentially caries-related microbes | *Abiotrophia; Campylobacter; Capnocytophaga; Cardiobacterium; Corynebacterium; Granulicatella; Kingella; Neisseria; Streptococcus; Veillonella* | Gross et al., 2012^5^ |
| Hydrogen sulfide producing microorganisms | *Bacteroides; Centipeda; Eubacterium; Fusobacterium; Peptostreptococcus; Prevotella; Selenomonas; Solobacterium; Treponema* | Basic et al., 2015^20^; Nani et al., 2017^21^; Persson et al., 1990^22^ |
| Nitrate reducing microorganisms | *Actinomyces; Brevibacillus; Fusobacterium; Granulicatella; Haemophilus; Leptotrichia; Neisseria; Porphyromonas; Prevotella; Rothia; Staphylococcus; Veillonella* | Doel et al., 2005^23^; Hyde et al., 2014^3^; |
| Microorganisms associated with *in vivo* or *in vitro* cortisol levels | *Acinetobacter; Actinomyces; Campylobacter; Cardiobacterium; Eikenella; Fusobacterium; Gemella; Granulicatella; Haemophilus; Lachnoanaerobaculum; Leptotrichia; Neisseria; Porphyromonas; Prevotella; Rothia; Selenomonas; Streptococcus* | Akcali et al., 2014^24^; Charalambous et al., 2024^11^; Jentsch et al., 2013^25^; Simpson et al., 2020^6^ |
| Microorganisms associated with cytomegalovirus titers | *Acinetobacter, Alysiella, Bradyrhizobium, Comamondaceae, Flavobacterium, Methylorubrum, Neisseria, Oxalobacteraceae, Sphingomonas* | Charalambous et al., 2024^11^ |
| Microorganisms potentially linked to stress or adversity exposure in humans | *Acinetobacter; Actinomyces; Aggregibacter; Aquabacterium; Capnocytophaga; Comamonas; Corynebacterium; Eikenella; Fusobacterium; Haemophilus; Lancefieldella; Leptotrichia; Neisseria; Porphyromonas; Prevotella; Rothia; Rothia; Schaalia; Solobacterium; Stomatobaculum; Streptococcus; Tanerella* | Alex et al., 2024^26^; Charalambous et al., 2021^10^; Gancz et al., 2024^13^; Huang et al., 2024^27^; Malan-Müller et al., 2024^9^; Nani et al., 2017^21^; Stoy et al., 2023^28^; |
| Microorganisms potentially linked to internalizing symptoms in humans | *Abiotrophia; Actinomyces; Alloprevotella; Bergeyella; Bulleidia; Butyrivibrio; Campylobacter; Cardiobacterium; Catonella; Centipeda; Cloacibacterium; Corynibacterium; Dialister; Eikenella; Filifactor; Fretibacterium; Fusobacterium; Gemella; Granulicatella; Haemophilus; Johnsonella; Kingella; Lachnoanaerobaculum; Lancefieldella; Lautropia; Leptotrichia; Ligilactobacillus; Megasphaera; Mitsuokella; Mobiluncus; Mogibacterium; Mycoplasma; Neisseria; Oribacterium; Parvimonas; Pauljensenia; Peptococcus; Porphyromonas; Prevotella; Pseudomonas; Schaalia; Selenomonas; Solobacterium; Stenotrophomonas; Stomatobaculum; Tanerella; Thermus; Treponema; Veilonella* | Al Bataineh et al., 2022^29^; Aleti et al., 2022^12^; Alex et al., 2024^26^ ; Gancz et al., 2024^13^; Levert-Levitt et al., 2022^30^; Malan-Müller et al., 2024^9^; Simpson et al., 2020^6^; Wingfield et al., 2021^8^; Ye et al., 2024^31^ |
|  | | |
| **Functional Features** | | |
| *Function/Description* | *Compound(s)* | *Citation(s)* |
| Aromatic amino acid metabolism | L-tryptophan; L-tyrosine ;L-phenylalanine | Aleti et al., 2022^12^; Lin et al., 2024^2^; Malan-Müller et al., 2024^9^; Paudel et al., 2022^16^; Qian et al., 2023^32^ |
| GABA metabolism | GABA | Malan-Müller et al., 2024^9^; Qian et al., 2023^32^ |
| Glutamate metabolism | L-glutamate | Malan-Müller et al., 2024^9^; Paudel et al., 2022^16^; Qian et al., 2023^32^; |
| Short-chain fatty acid metabolism | Butanoate; propanoate; acetate | Lin et al., 2024^2^; Malan-Müller et al., 2024^9^; |
| Hydrogen sulfide metabolism | Hydrogen sulfide | Lin et al., 2024^2^ |
| Nitrate metabolism | Nitrate, nitrite | Shannon et al., 2022^4^ |
| Lippopolysaccharide pathways | All instances of class “a lipopolysaccharide” or “an o-antigen” | Mamunur et al., 2023^33^; Wang et al., 2019^34^; Xue et al., 2022^17^ |
| Peptidoglycan pathways | All instances of class “a peptidoglycan” | Paudel et al., 2022^16^ |
| Trimethylamine n-oxide | Trimethylamine n-oxide | Brunt et al., 2020^35^ |
| A peptide antibiotic | All instances of “a peptide antibiotic” | Zhao et al., 2023^36^ |
|  |  |  |
| Taxonomic features are selected at the genus level from the citations listed, as described above. Where a publication lists a feature at a more granular level (i.e. amplicon sequence variant), its genus is used in this module. Features at a higher level than genus are not used in this module. Functional features are derived by selecting compounds, listed above, in the MetaCyc^15^ database and using the SmartTables feature to identify all pathways in which that compound is an input, output, or intermediate. For lipopolysaccharides and peptidoglycan, all instances of those classes were used in this manner. | | |
|  | | |

**Supplement 3: Microbiome relationships with chronic stress, adiposity, and time of day**

*Statistical Analyses*

Data collection and preprocessing are described in the main text. We used ordinary least squares regressions to test whether PSS scores (chronic stress), waist to height ratio (WHtR), or time of day of study participation explained variance in alpha diversity (Faith’s or Shannon’s diversity), genus-level abundance, or PICRUSt2 estimated functional features. We used Benjamini-Hochberg multiple comparison with a false discovery rate of .250 to correct for multiple comparison on the explanatory variables of interest. Each predictor of interest (PSS, WHtR, or time of day) and microbiome feature type (alpha diversity, genus-level abundance, or functional feature abundance) comprised a family of models for the purpose of multiple comparison correction, yielding 9 model families in total. All analyses controlled for CTQ, OHQ, and sugar consumption.

*Results*

Neither PSS (Table S2) nor WHtR (Table S3) were significantly associated with either alpha diversity metric, nor with any nor PICRUSt2 estimated functional features. Time of day was significantly associated with Shannon’s alpha diversity, and we therefore decided to conduct supplementary analyses to test whether any significant results involving associations with Shanonn’s diversity were robust to controlling for time of day. However, we saw no significant associations with Shannon’s diversity in the primary analyses, and thus we determined that no supplementary analyses to control for time of day were needed.
